# Alder pollen of *Alnus spaethii* at christmas: from epidemiology of molecular allergens to the political solution

**DOI:** 10.1186/2045-7022-4-S2-P36

**Published:** 2014-03-17

**Authors:** Markus Gassner, Regula Gehrig, Peter Schmid-Grendelmeier

**Affiliations:** 1 University Zurich, Spitalstr. 8, Grabs, CH-9472, Switzerland; 2 MeteoSwiss, Aerobiology, 8044 Zurich, Switzerland; 3 Zurich University Hospital, Allergy Unit, 8091 Zürich, Switzerland

## Background

A newly planted alley with hybrid trees (Alnus x spaethii: A. japonica x A. subcordata) changes the exposition, emission and sensitization pattern in a population. This by hybridizing man made and man planted tree is recommended to plant in towns. We report about the political consequences.

## Methods

IgE antibodies to 103 molecular allergens were measured (using ImmunoCAP ISAC) in serum samples obtained from 54 students in 1986 and from 46 students in 2006. In 2010, 12 of the former students from 1986 with positive IgE antibodies to inhalant allergens where retested. Along the main boulevard in Buchs 96 hybrid trees (Alnus spaethii) were planted 1995-2000. Atmospheric pollen levels were measured in Buchs since 1984, phenological observation since 2009.

## Results

IgE antibodies against the main allergen of alder trees (rAln g 1), which were not detected in any child in 1986, were found in 10.9% of unselected schoolchildren in 2006. This increased prevalence of sensitization was not seen with pollen of other trees, such as birch, hazel, ash, or plane. Among the 12 former students who were tested at the ages of 15 and 39 years, 3 (25%) showed newly detectable IgE antibodies to alder pollen in 2010. Although none of the schoolchildren reported having allergic symptoms in December from 1983 through 1986. Phenological observations since 2009 show constantly an early flowering of A. spaethii between Christmas and New Year's day, as fare two months earlier as the indigenous alder (A. glutinosa, A. incana). 236 gr of pollen were directly harvest at 27.12.2011 from some branches of a tree. All of the alder trees in this street had emitted tonnes of pollen every year. A. spaethii trees produce much more pollen than indigenous species.

## Conclusions

Alnus spaethii pollen induced allergic sensitizations and symptoms as rhinoconjunctivitis in winter with a much higher emission of pollen than indigenous alder species. It remains unclear why this tree is flowering so early every year (effect of climate, chilling, street lighting). These alder trees are even now replaced by amber trees (Liquidambar styraciflua worplesdon) because of their too fast growth, too much shadow and more littering problems of leaf and fruits as well as the described allergological problems. Plant trees in towns, but consider also the allergenic potential of their pollen.

